# Ductal aneurysm masquerading as nonresolving pneumonia: A challenging differential!

**DOI:** 10.4103/0974-2069.64364

**Published:** 2010

**Authors:** Maitri Chodhary, Munesh Tomar, Sitaraman Radhakrishnan

**Affiliations:** Department of Congenital and Pediatric Heart Diseases, Escorts Heart Institute and Research Center, New Delhi, India

**Keywords:** Ductal aneurysm, PDA, pneumonia

## Abstract

We report here, the case of a six-and-a-half-month-old boy investigated for persistent respiratory distress and homogeneous opacity in the left upper lobe. Echocardiography revealed a giant ductal aneurysm compressing the left pulmonary artery and upper lobe division of the left bronchus. Computerized tomography angiogram delineated the exact anatomy and prompt surgical resection provided a successful cure to this lesser known entity.

## CASE REPORT

**Figure 1:**
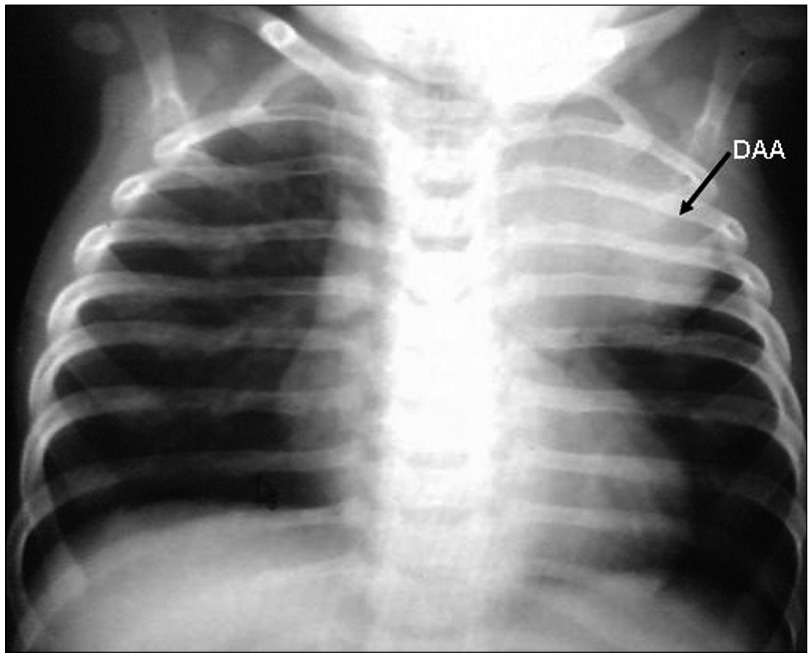
Chest X-ray (PA view) showing homogenous rounded opacity in the left upper thorax produced by the ductal aneurysm, normal lung parenchyma, normal pulmonary blood flow, and there was no cardiomegaly. DAA-ductus arteriosus aneurysm

S.G., a six-and-a-half-month-old boy, first in birth order, and product of a full-term normal delivery, was born with a weight of 2.5 kg, with bilateral congenital talipes equinovarus. Anomaly scanning including a neonatal chest X-ray failed to detect any other organ involvement. He was apparently well till one month of age and then developed persistent respiratory distress, failure to thrive, and had three episodes of 'pneumonia'. A chest X-ray revealed homogenous opacification of the whole left upper lobe [Figure 1], which was treated as collapse-consolidation. However, there was no clinical or radiological response to multiple courses of antibiotics. In view of the 'non resolving pneumonia', computed tomogram (CT) of the chest was done and a diagnosis of aneurysm from descending aorta and left upper lobe collapse-consolidation was made. He was referred to our institute for further evaluation.

On examination, the child weighed 5.6 kg (74% of expected) and was tachypneic (respiratory rate 58/minute with intercostal and subcostal retractions). Cardiovascular examination revealed a normal cardiac size, normal heart sounds, grade 2/6 short ejection systolic murmur at the pulmonic area, and no evidence of congestive cardiac failure. All peripheral pulses and blood pressure were normal. Interestingly an expansile mass was seen in the suprasternal notch during crying.

EchoDoppler examination performed using the Sonos 7500 machine with broadband transducers (S8 and S 12) in the subcostal, apical, parasternal, and suprasternal views showed no structural heart defect and normal ventricular dimensions and function. In the parasternal long axis view, the ascending aorta was dilated (Z score + 4.4). The aortic valve was tricuspid. Suprasternal long axis and short axis views showed a large ductal aneurysm rising from the descending aorta, distal to the left subclavian artery. The ampulla of this aneurysm was wide open (7 mm in size), but the pulmonary end was restricted and there was a tiny patent ductus arteriosus with a left-to-right shunt [Figure 2a‐c]. There was no tricuspid regurgitation and the patent ductus arteriosus (PDA) flow was insignificant, which could not be aligned to assess pulmonary artery pressure. There was spontaneous echo contrast inside the aneurysm, without any thrombus. This was compressing the left pulmonary artery. A CT angiogram confirmed the huge aneurysm rising from the ampullary end of the ductus, with compression of the left pulmonary artery and upper lobe division of the left bronchus. In addition, there was compensatory hyperinflation of the rest of the left lung [Figure 3a‐c].

**Figure 2a:**
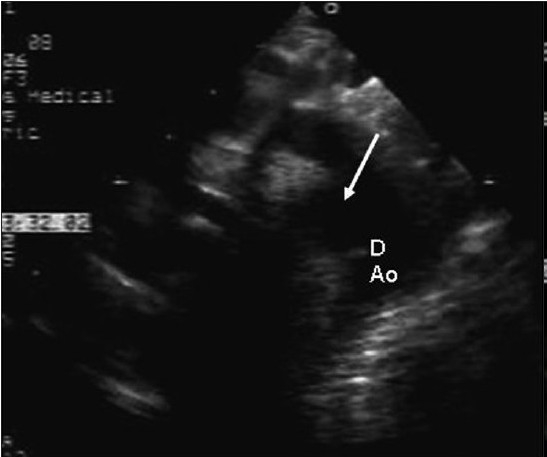
Two-dimensional echocardiography from modified suprasternal echocardiographic long axis view showing the aneurysm and the ampulla (arrow)

**Figure 2b:**
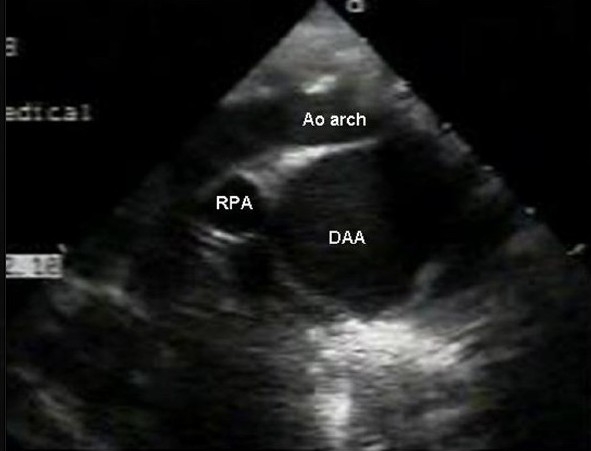
Two-dimensional echocardiography from modified suprasternal short axis view showing large ductal aneurysm with wide ampulla

**Figure 2c:**
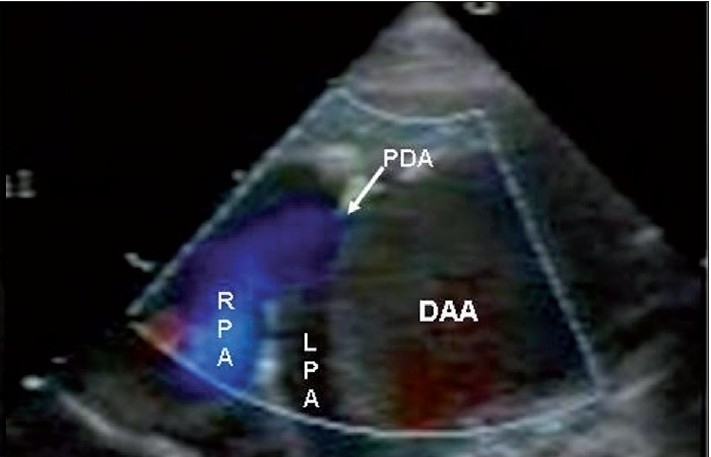
High parasternal short axis view with color flow mapping showing tiny ductal flow (left to right) with normal flow in the right pulmonary artery DAA-ductus arteriosus aneurysm, RPA-right pulmonary artery, LPA-left pulmonary artery, DAo-descening aorta. Ao arch-aortic arch, PDA-patent ductus arteriosus

**Figure 3:**
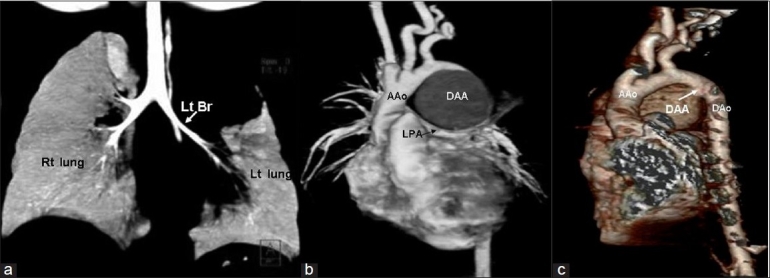
High resolution computed tomography (HRCT) angiography of the chest (a) Compression of left upper lobe bronchus and left upper lobe collapse (b) Large ductal aneurysm compressing the left pulmonary artery (c) Three-dimensional reconstruction of HRCT chest from the posterior aspect, showing the large ductal aneurysm originating from the descending aorta just distal to the origin of the left subclavian artery. Lt Br-left bronchus, rt lung-right lung, lt lung-left lung, AAo-ascending aorta, Dao-descending aorta, LPA-left pulmonary artery, DAA-ductus arteriosus aneurysm

Preoperatively a ductal aneurysm of size 3 cm × 3 cm arising from descending aorta, just distal to the origin of the left subclavian artery, was seen. Under cardiopulmonary bypass, the pulmonary end of the ductus was ligated and then the aortic end was repaired with an autologous pericardium and the wall of the aneurysm was excised partially, while protecting the phrenic and recurrent laryngeal nerves. Postoperative follow-up at the end of two years showed complete regression of the aneurysm with laminar flow into the left pulmonary artery and descending aorta, on echocardiography.

## DISCUSSION

A ductus arteriosus aneurysm is a rare lesion characterized by localized saccular or tubular dilatation of the ductus arteriosus. A PubMed search revealed 144 cases; 106 spontaneous and 38 postoperative.[1] The published Indian experience is limited to 16 cases (youngest patient was four years old).[2] Spontaneous aneurysm is seen most commonly in children less than two months of age. The etiology may be congenital, post infectious or associated with connective tissue disorders (Smith-Lemli-Opitz, Marfan's, Ehlers-Danlos, and Larsen syndromes). Most of the spontaneous aneurysms in literature have been diagnosed incidentally at autopsy; in a later era, transthoracic and transesophageal echocardiography has been very useful in diagnosing and monitoring the aneurysm.

Two different theories of pathogenesis have been proposed. Dr. Helen Taussig postulated that the primary event is failure of the duct to close at the aortic end after the pulmonary end has closed. This, along with concomitant exposure to systemic arterial pressure, causes aneurysmal dilatation. This observation is supported by the fact that 30% of the infantile ductal aneurysms are closed at the pulmonary end; the second theory suggests that congenital or acquired structural weakness of the ductal wall, such as reduced intimal cushion formation or abnormal deposition of elastin and glycoproteins, are responsible.[3]

Our case supports this theory, as the pulmonary end was patent and the ascending aorta was also dilated. The presence of talipes and the unexplained dilated aortic root could be markers of connective tissue disorder. Our patient did not meet the required diagnostic criteria for any specific syndrome. Karyotyping was normal and so we cannot really comment on whether the concomitant presence of ductal aneurysm with talipes and dilated ascending aorta was a chance association or a hitherto unknown genetic syndrome.

Because of the rarity and atypical presentations, often this diagnosis is missed. We feel that any infant with persistent lung collapse/consolidation should undergo echocardiography to exclude congenital heart disease. In young infants like our patient, conventional transthoracic echocardiography provides excellent images. Transesophageal echo, CT, magnetic resonance imaging (MRI) and Aortography may be required in the older age group.[4] Preoperative CT angiogram with 3D reconstruction helped the surgeon to formulate the exact surgical plan.

Spontaneous rupture, erosion, thromboembolism, and compression of the adjacent structures are the feared complications occuring in as high as 31% of symptomatic infants less than two months. Surgery is recommended if a significantly large aneurysm does not regress spontaneously within a short follow up.[5] In our patient, there was compression of the left pulmonary artery and ipsilateral bronchus by the huge aneurysm and early surgery was justified.

## CONCLUSION

We report this rare manifestation of ductal aneurysm and hope to increase the awareness among general pediatricians, radiologists, and echocardiographers to this possibility. Common investigations help in the complete diagnosis. Surgical intervention at an appropriate time can result in complete cure. However, these patients should also be screened for associated genetic and connective tissue disorder and detailed evaluation is mandatory.
